# Analysis of Nucleosides and Nucleotides in Plants: An Update on Sample Preparation and LC–MS Techniques

**DOI:** 10.3390/cells10030689

**Published:** 2021-03-20

**Authors:** Henryk Straube, Claus-Peter Witte, Marco Herde

**Affiliations:** Department of Molecular Nutrition and Biochemistry of Plants, Leibniz Universität Hannover, 30419 Hannover, Germany; straube@pflern.uni-hannover.de

**Keywords:** nucleotides, nucleosides, mass spectrometry, liquid chromatography, quenching, solid phase extraction, hydrophilic interaction liquid chromatography (HILIC), plant nucleotide metabolism, plant metabolomics

## Abstract

Nucleotides fulfill many essential functions in plants. Compared to non-plant systems, these hydrophilic metabolites have not been adequately investigated in plants, especially the less abundant nucleotide species such as deoxyribonucleotides and modified or damaged nucleotides. Until recently, this was mainly due to a lack of adequate methods for in-depth analysis of nucleotides and nucleosides in plants. In this review, we focus on the current state-of-the-art of nucleotide analysis in plants with liquid chromatography coupled to mass spectrometry and describe recent major advances. Tissue disruption, quenching, liquid–liquid and solid-phase extraction, chromatographic strategies, and peculiarities of nucleotides and nucleosides in mass spectrometry are covered. We describe how the different steps of the analytical workflow influence each other, highlight the specific challenges of nucleotide analysis, and outline promising future developments. The metabolite matrix of plants is particularly complex. Therefore, it is likely that nucleotide analysis methods that work for plants can be applied to other organisms as well. Although this review focuses on plants, we also discuss advances in nucleotide analysis from non-plant systems to provide an overview of the analytical techniques available for this challenging class of metabolites.

## 1. Introduction

**Figure 1:**
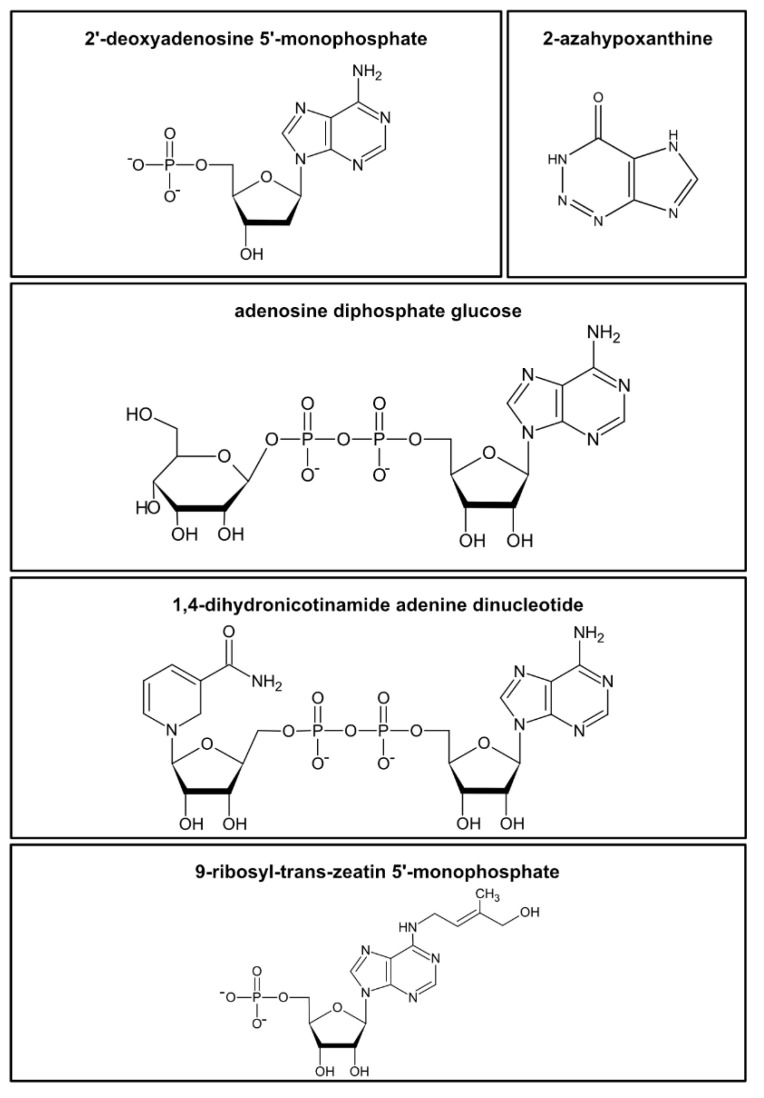
Examples of the diversity of nucleotides and structurally related metabolites in plants. Examples are shown for a nucleotide monophosphate (2′-deoxyadenosine 5′-monophosphate), a “fairy chemical” (2-azahypoxanthine), a nucleotide sugar (adenosine diphosphate glucose), a nucleotide cofactor (1,4-dihydronicotinamide adenine dinucleotide, NAD), and a cytokinine-ribotide (9-ribosyl-trans-zeatin 5′-monophosphate).

Nucleotides (NTs), nucleosides (Ns), nucleobases (Nbs), and many derived compounds, for example, nucleotide sugars and nucleotide-containing cofactors, are central metabolites in all organisms (Figure 1). The metabolism of nucleotides in plants and their physiological functions, including those beyond being building blocks of nucleic acids, were recently reviewed [1]. For the investigation of nucleotides in plants, the availability of methods for their comprehensive analysis and robust quantification in plant extracts is pivotal. In this review, we summarize the state of the art for NT and N analysis in plants and also discuss methods used in other organisms that may be applicable for plants. Arguably, the most powerful equipment for metabolite analysis is a chromatographic separation system coupled to an electrospray ionization (ESI) mass spectrometer (MS). This technology is in the focus here. However, our recent study [2] also emphasized the importance of sample preparation for comprehensive NT and N analysis; therefore, we discuss the entire workflow of NT and N analysis in all its aspects—from sample preparation to mass spectrometry—and we comment on how the different steps of the workflow influence each other.

The choice of analytical strategies is naturally determined by the physicochemical properties of NTs and Ns. These are polar compounds and the phosphate group(s) of the NTs deprotonate and carry negative charges (pKa values of about 2) [3] within a broad range of pH values. NTs and Ns contain primary, secondary, and tertiary amines, which, especially under more acid conditions, can protonate and acquire a positive charge especially under more acidic conditions [3]. Due to these characteristics, Ns and especially NTs are better soluble in polar (aqueous) solvents.

The concentrations of different NTs vary significantly in plant cells. For example, ribonucleotide triphosphates are about 1000-fold more abundant than deoxyribonucleotide triphosphates [2]. To detect nucleotides of low abundance, additional techniques for enrichment are usually required.

We noticed that methods for nucleotide analysis that are well established in some non-plant systems are not suitable for plants. For example, the simple workflow we applied for the analysis of deoxyribonucleotides (dNTs) in *Drosophila* [4] failed to work for samples from *Arabidopsis thaliana*. For the detection of low-abundance nucleotides such as dNTs in phylogenetically diverse plant species, the development of a more complex method was necessary [2]. We can only speculate why the analysis of plants is more complicated, but one contributing factor may be the complexity of the plant matrix which contains a plethora of secondary metabolites [5]. These are probably the reason for the strong ion suppression effects in the ESI source, called matrix effects (ME), which decrease the sensitivity and are usually observed when analyzing plant extracts [2]. Thus, a highly sensitive method for the analysis of the plant NT and N metabolome must include steps to separate these from other interfering metabolites before the chromatographic step. However, if only the more abundant ribonucleosides (rNs) and ribonucleotides (rNTs) are to be monitored, methods for the analysis of the polar metabolome that do not require such extensive sample preparation are sufficient [6,7,8]. For many years, distinct analysis methods for the NT and N metabolome have been evaluated and improved. For a brief overview of the milestones in plant nucleotide analysis, see Table 1. In the following sections, we discuss the workflow for modern NT/N analysis step by step.

## 2. Disruption of the Tissue

**Figure 2:**
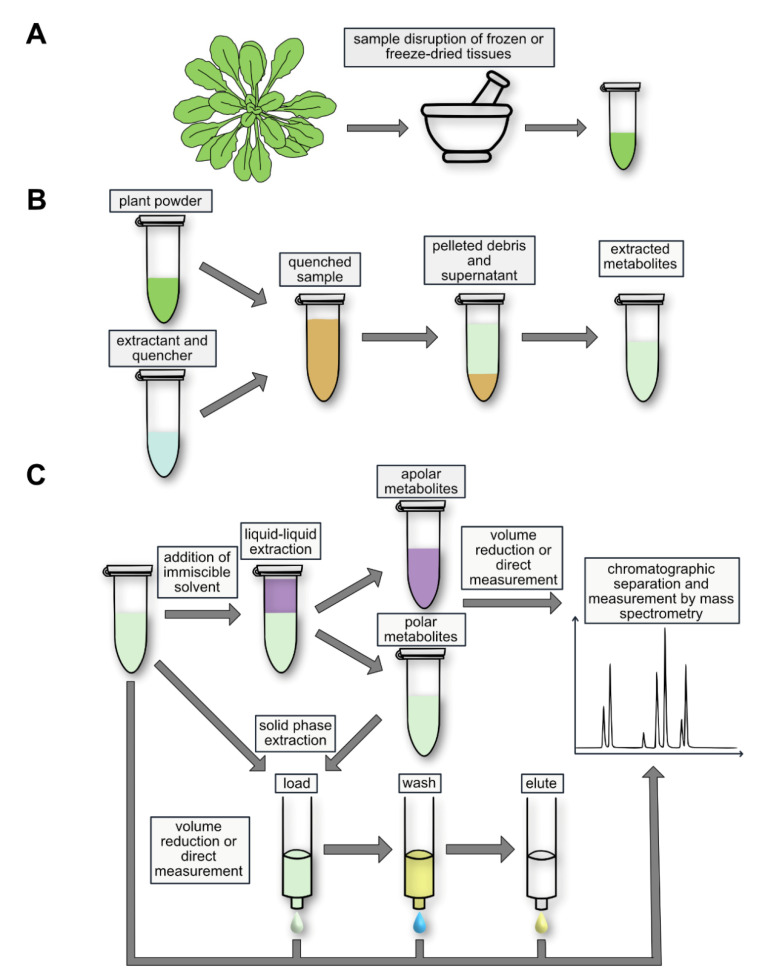
Schematic representation of a typical workflow for the analysis of nucleotides and nucleosides in plants. (**A**) Tissue disruption, here symbolized by mortar and pestle, is followed by (**B**) quenching of the sample and extraction of the metabolites. (**C**) Subsequently, non-polar and polar metabolites are separated by liquid–liquid extraction (LLE). The fraction containing polar metabolites can be further purified to enrich for nucleotides by solid-phase extraction (SPE) with a weak anion-exchange column. In this procedure, nucleotides are separated from nucleosides that are present in the flow-through. Extracts containing nucleosides and nucleotides can be separately analyzed using liquid chromatography and mass spectrometry.

Because plant cells have a cell wall, efficient physical disruption is required, often using a mortar and pestle or an oscillating mixer mill (Figure 2A). The NTs and Ns are best quantified by the isotope dilution technique [2,15,16]. This accounts for analyte losses in the workflow after tissue disruption, as well as the matrix effect (see later). The efficiency of the tissue disruption itself cannot be assessed because the isotope standards are added to the extraction buffer. Incomplete tissue disruption can, therefore, cause an underestimation of the actual analyte concentration in vivo and result in an increased standard deviation because the degree of disruption is usually not reproducible.

Tissue disruption mixes metabolites and enzymes from all cellular compartments, which can result in metabolite instability, for example, because cytosolic NTs are dephosphorylated by vacuolar phosphatases. It is, therefore, crucial to stop all enzymatic activities when the cellular integrity is lost. This is usually achieved by deep-freezing the sample during tissue disruption with liquid nitrogen. Alternatively, the sample may first be freeze-dried before grinding it to a fine powder in a dry state at room temperature. In our hands, the NT extraction with an aqueous solvent is similarly efficient for freeze-dried material of *Arabidopsis thaliana* compared to frozen fresh material (our data, unpublished), which is consistent with data from yeast [17]. Enzymes may be reactivated when frozen or freeze-dried samples thaw or come in contact with an aqueous extraction buffer.

## 3. Quenching of the Sample

Metabolomic measurements often attempt to estimate the pool sizes of metabolites in vivo. This is only possible if enzymatic processes that can alter metabolite concentrations are irreversibly suppressed (quenched) during and after extraction (Figure 2B). Non-polar metabolites are frequently extracted with mixtures of a high proportion of an organic solvent and some water. Such extractants efficiently quench the sample by precipitation of proteins. However, for NT extraction, organic–aqueous mixtures are problematic because it has been noted that phosphatases, which can dephosphorylate NTs, are not fully quenched in such solvents [5,18,19,20]. In addition, methanol or ethanol applied cold or boiling can lead to the formation of methyl or ethyl phosphate, which might be derived from nucleophilic attack of the alcohol on the anhydride bonds of the NTs [5,18,21]. Rapid boiling of the ground sample in aqueous buffers without alcohols can be used for Ns [22], but this method is also not suited for NT analysis [2,23]. However, if organic–aqueous mixtures or water are to be used, phosphatases can be inhibited by sodium fluoride [12]. Nonetheless, the effectiveness of the inhibitor should be tested.

A very efficient method for quenching is protein precipitation by addition of a strong acid [5,9,23,24,25]. Frequently, perchloric acid (PCA) or trichloroacetic acid (TCA) is used. A disadvantage of PCA is that it precipitates upon neutralization which may cause some loss of analytes, especially in samples with high protein content [2,26]. TCA can be removed and neutralized by liquid–liquid extraction (LLE) using a trioctylamine-containing organic phase that is immiscible with water [10]. Drawbacks of this method are the additional hands-on time and potential chemical alterations of metabolites when kept at extreme pH [5]. However, swift handling and cold temperature lead to good recovery rates using TCA coupled to an LLE. The LLE additionally removes undesired non-polar metabolites, further reducing matrix complexity [2]. For all quenching methods, especially those using harsh conditions (such as heat or low pH), it is mandatory to determine whether NTs and Ns are quantitatively recovered and not chemically modified or degraded by the procedure.

## 4. Extraction of NTs and Ns from Plant Samples

Good solubility of the metabolites in the extraction solvent is crucial for efficient extraction. NTs and Ns are polar and, at least in the case of NTs, also charged, requiring a polar solvent for efficient extraction (Figure 2B). Often the extractant is aqueous [2,22], but water/acetonitrile mixtures are in use as well [27,28,29]. The solubility of the analytes in the solvent should be considered when choosing the extractant volume in relation to the sample amount, especially when less soluble Nbs [3] are extracted. If solubility is limiting, re-extraction of a pellet will result again in high analyte concentration in the supernatant. The solubility is also dependent on the pH of the extractant. For the extraction of polar metabolites, a 1:10 ratio of fresh material to solvent is recommended, whereas a ratio of 1:100 is recommended for lyophilized material [30].

Extraction and all following steps should be designed to stabilize the analytes. This is essential for NTs, since they may lose a phosphate moiety, for example, during chromatography [31]. One study found that acidification of the extractant can substantially improve the stability of NTs [27]. Our recent work confirmed that, at least for the duration of the extraction, NTs and Ns are acid-stable with good recoveries [2]. Although, more studies have used acidic conditions for NT extraction, there are protocols for the determination of rNTs in *Nicotiana tabacum*, *Arabidopsis thaliana*, and *Solanum lycopersicum* using quenching with a high pH, which also led to excellent recoveries [32]. At high pH, NTs were reported to be stable even when stored for 3 days at room temperature [32]. In general, frozen nucleotide solutions are stable for a prolonged time if the acid from the quenching step is removed or neutralized [33]. With methods that directly use clarified crude extracts for MS analysis, extraction may result in the release of metabolites that cause ion suppression and, thus, decrease the sensitivity in mass spectrometry (matrix effect) [27]. A further concern is that the concentrations of NTs and Ns may be overestimated if the extraction procedure can potentially release them from other metabolites. The most obvious example is the hydrolytic release of phosphate groups from nucleotide triphosphates (NTPs), reducing NTP concentrations and leading to an overestimation of nucleotide diphosphates (NDPs), nucleotide monophosphates (NMPs), or Ns. Another example is the release of uridine diphosphate (UDP) from UDP-glucose, since the hydrolysis of this compound was observed under mild acidic conditions and at an elevated temperature in nucleotide sugar analysis [34].

Adding a non-polar solvent immiscible with water to the extraction will result in the partitioning of Ns and NTs to the polar/aqueous phase and of non-polar metabolites to the non-polar phase (Figure 2C). Separation and individual processing of both phases results in a coarse fractionation of metabolites which is used in studies simultaneously reporting polar and non-polar metabolites [35]. As a non-polar solvent immiscible with water, methy-*tert*-butyl-ether (MTBE) is a less toxic alternative to fluorocarbons such as 1,1,2-trichlor-1,2,2-trifluorethan used previously [10] and is, therefore, more environmentally friendly and safer to handle [35]. Moreover, dichloromethane and water-saturated diethyl ether have been used as less toxic alternatives [2,33]. Hexane or chloroform was employed in some studies for the removal of phospholipids by LLE [36,37]. The addition of polyvinylpolypyrrolidone can remove phenolic compounds that cannot be eliminated by other means and can interfere with the detection of NTs [11,38].

The rNTs and rNs, but not their dNT and deoxyribonucleoside (dN) counterparts, have two neighboring hydroxyl groups (*cis*-diol) in the sugar moiety. This feature can be used either for the extraction of rNTs and rNs (see next chapter) or for their selective degradation by periodate and methylamine-driven *β*-elimination [11,39,40,41]. Selective degradation of the more abundant rNTs and rNs can enhance the detection of dNs and dNTs by reducing the specific NT/N background. This can be explained, for example, by less competition for binding to the resin during solid-phase extraction (next chapter) or simply by less ion suppression in the ESI source. Selective *cis*-diol degradation may also be an improvement for detecting NTs and Ns with modified sugars such as 2′-*O*-methylguanosine, because these will be protected from degradation. When using the selective degradation protocol, it has been recommended to first determine the recovery rates of the metabolites in focus, because, even though dNTs are generally not degraded, deoxyguanosine triphosphate (dGTP), for example, can react with periodate [42].

## 5. Solid-Phase Extraction

Solid-phase extraction (SPE) is a cartridge chromatographic technique where the interplay between a solid phase of certain binding characteristics and a mobile liquid phase for washing and elution is important to obtain the desired separation results (Figure 2C). The advantage of using SPE is that the analytes of interest are separated from unwanted matrix and are potentially concentrated on the resin. Both effects increase the sensitivity for the analytes in the mass spectrometer. A disadvantage of SPE is that it is laborious to set up. Different solid and mobile phases must usually be tested, and careful adjustment of all parameters such as sample volume and composition of the mobile phases for loading, washing, and elution is required.

For the analysis of NTs and Ns from plants and algae, we recently described a protocol employing a mixed-mode SPE, which is a weak anion-exchange resin together with a hydrophobic binding component [2]. The NTs were retained, but the uncharged Ns did not bind to the resin and were recovered in the flow-through. Choosing the pH of the mobile phase is important because it can influence the charge of metabolites and, therefore, their retention on the SPE cartridge. To facilitate the choice of the pH, a database of pKa values [43] (http://ibond.nankai.edu.cn, accessed on 18 March 2021) for a variety of metabolites is available. On a weak ion-exchange resin, changing the mobile phase pH is used to elute metabolites, because the pH shift causes a loss of ionization of the stationary phase [44]. The elution in our protocol is based on this principle. The ionic strength of the mobile phase is also an important determinant for retention on an ion-exchange SPE cartridge. We observed that the less charged NMPs were not fully retained when a mobile phase of higher ionic strength was used, whereas NTPs were still bound quantitatively [2]. This result indicates that further sub-fractionation of the NT metabolome by fine-tuning the ionic strength of the mobile phase may be possible.

Ion exchange has also been employed in other protocols for sample preparation in NT analyses. For example, 23 NTs and cofactors from *Arabidopsis thaliana* samples were isolated by a combination of cation exchange and weak anion exchange [13]. An anion-exchange resin was also applied for magnetic dispersive solid-phase micro extraction of NTs from medicinal plants (*Anoectochilus roxburghii*) in a batch procedure. The surface area of this resin is very high potentially increasing the metabolite yield [44]. Furthermore, a mixed-mode anion-exchange sorbent was used as stationary phase to extract Ns, Nbs, and cytokinins from *Physcomitrella patens* [14].

Apart from ion exchange, other separation principles were also applied. Graphitized carbon as a stationary phase for the SPE of polar compounds has gained popularity [45]. In particular, nucleotide sugars can be successfully extracted with this method using an ion-pairing reagent as eluent [46,47,48]. Stationary phases with phenyl-boronate groups are uniquely suited to retain sugars with a *cis*-diol group (see Section 4), which can be used not only for the removal or enrichment of rNTs and rNs [49], but also for the extraction of brassinosteroids from *Arabidopsis thaliana* [50]. A recently developed strategy uses molecularly imprinted polymers to provide tailor-made binding sites for binding specific metabolites to a stationary matrix [51]. By this approach, modified Ns and NTs have been extracted from urine to serve as biomarkers [52,53]. The discovery of so-called fairy chemicals in several plants (*Arabidopsis thaliana, Oryza sativa, Solanum lycopersicum*; 2-azahypoxanthine, imidazole-4-carboxamide, and 2-aza-8-oxohypoxanthine [54,55]), which are structurally closely related to Nbs and Ns, involved a fractionation by flash chromatography [56] that is based on a silica stationary phase also known from thin-layer chromatography [57,58]. Once identified, it was shown that these metabolites can also be enriched from a complex matrix by a combination of two different SPE procedures. A so-called hydrophilic–lipophilic balance SPE was followed by a cation-exchange SPE that removed undesired compounds and fractionated the fairy chemicals by using basic and acidic eluents [59].

## 6. Derivatization

**Figure 3:**
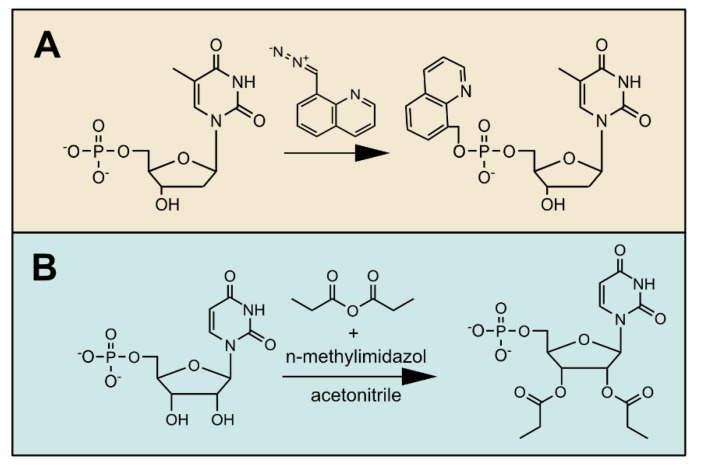
Examples of different derivatization strategies for nucleotides. (**A**) 8-(diazomethyl) quinoline reacts with the phosphate group neutralizing a negative charge; (**B**) propionic anhydride is used for the derivatization of hydroxyl groups. Both strategies reduce the polarity of the nucleotide and facilitate chromatographic separation, which fosters a more sensitive detection by mass spectrometry.

Chemical derivatization uses chemical agents that react with the targeted metabolites and form a predictable product that is quantified instead of the original metabolite (Figure 3). Often, the aim of derivatization is to mask the polar features of a metabolite, which facilitates chromatographic separation and/or detection by mass spectrometry [60]. For NTs, a derivatization protocol with 8-(diazomethyl)quinoline was reported recently. This chemical reacts with the phosphate groups of NTs, forming a product that is less polar (Figure 3A). Such derivatives can be detected with higher sensitivity, even allowing the quantification of rare modified NTs in mammalian cell extracts [61]. In *Medicago truncatula*, this derivatization strategy was used for the sensitive detection of sugar phosphates [62]. Since the derivatization reagent is highly reactive and unstable, the success of this strategy depends in part on the concentration of other phosphorylated metabolites that consume the reagent and, thus, relies on the efficiency of other clean-up steps in the workflow [62].

Another useful derivatization strategy is the esterification of the hydroxyl groups of the sugar moiety with propionic anhydride (Figure 3B), which was used to detect cytokinins and NTs in *Arabidopsis thaliana* with high sensitivity [60]. A very similar approach employing acetic anhydride was used to monitor cytokinins by gas chromatography [63]. Silylation, often used in the context of gas chromatography, can also foster the detection of NTs by LC–MS [64].

## 7. Reduction in Sample Volume

The solvent is often removed to concentrate the sample or to change from a polar to a non-polar solvent prior to the injection in a chromatographic system. If polar metabolites have been extracted, this usually involves the evaporation of an aqueous phase, for example, in a speed vacuum centrifuge. Because of the high boiling point of aqueous solvents, solvent evaporation is a comparatively long procedure that exposes the sample to ambient temperatures for an extended period of time. Degradation of labile compounds might, therefore, be a problem, which can also be accelerated by possible pH changes during the evaporation of the solvent.

For NT analysis, we tested freeze-drying and vacuum centrifugation of samples. Freeze-drying has the advantage that the samples are cooled during the procedure; however, with vacuum centrifugation, which requires about 2 h at room temperature, we also obtained satisfying recoveries of NTs [2]. Organic–aqueous mixtures such as water–methanol for polar metabolites (used, for example, in [35]) are often incompatible with freeze-drying equipment. Such extracts must be dried down in a nitrogen flow evaporator or a speed vacuum centrifuge. The dried pellets are then dissolved in polar or non-polar solvents depending on the type of chromatography that follows. The sample volume and the composition of the injection solvent are critical for the chromatographic separation. For an HILIC (hydrophilic interaction liquid chromatography) stationary phase, the proportion of organic solvent in the injection solution has been optimized to ensure maximal solubility of NTs without compromising the chromatography [31].

## 8. Chromatographic Separation

Sample preparation techniques such as LLE and SPE greatly reduce the complexity of a sample. The chromatography then separates the sample analytes from each other so that they ideally arrive at different times at the ESI source of the MS. Together, these techniques ensure that the MS processes as few metabolites/ions as possible at any given time. This is important because it greatly improves the sensitivity (see next section). Furthermore, the unequivocal identification of analytes benefits from sample preparation and especially chromatography. For example, naturally occurring heavy isotopes of adenosine monophosphate (AMP) cannot be distinguished from inosine monophosphate (IMP) with a triple-quadrupole mass spectrometer. This is because they have nearly the same mass (i.e., they are isobaric), and such compounds often have even identical fragmentation patterns [65]. It is, therefore, essential that they are chromatographically separated and reach the MS at different times.

Basic concepts of liquid chromatography were reviewed elsewhere [66]. Here, we would like to focus on systems that are especially useful for the analysis of NTs and Ns. The well-established reverse-phase chromatography uses non-polar stationary phases, for example, C8 or C18 columns, which are not able to retain Ns and NTs. Modifications of such hydrophobic stationary phases by adding polar groups—either at the silanol groups (end-capping) or directly embedded as ligands—result in the increased retention of polar and decreased retention of non-polar metabolites [67,68,69]. For the separation of Ns, for example, the non-canonical dNs [70], such matrices have been used successfully, but the retention of NTs is usually poor. This problem can be addressed by adding an ion-pairing agent to the mobile phase, which promote the retention of NTs on a hydrophobic stationary phase. However, the classical ion-pairing agents are not volatile and contaminate the source of the MS. They are also associated with suppression of the signal [71,72]. Interestingly, the development of volatile ion-pairing agents with favorable characteristics for mass spectrometry recently increased the interest in reverse-phase chromatography for the separation of charged compounds [73], and applications for NTs also exist [74].

A stationary phase, especially useful for the analysis of polar and charged metabolites, is porous graphitized carbon (PGC). Such a matrix separates polar and charged substances in a reverse-phase mode fully compatible with mass spectrometry. The main disadvantage is a certain instability of retention times which may hamper the identification of compounds, especially in non-targeted metabolome studies. It was reported that the redox status of the column can interfere with retention times [12]. In our recent study, we used PGC chromatography for NT and N analysis [2] and did not observe marked retention time instability. Although we did not address this formally, we found that samples processed by SPE ran more reproducibly on PGC chromatography than unprocessed samples. Generally, thorough column equilibration seems to improve retention time stability. PGC chromatography is a powerful tool for NT and N analysis [2], which can also separate isobaric 3′- and 5′-nucleotide monophosphates (our data, unpublished). As mentioned above, the chromatographic separation of isobaric NTs is very important to avoid that metabolites of nearly identical mass are wrongly assigned and quantified, which could lead, for example, to confounding dGTP with adenosine triphosphate (ATP) [12] or IMP with AMP isotopes. Furthermore, several publications have shown that PCG-chromatography is able to separate nucleotide sugars [12,46,47].

An alternative separation technique for the polar metabolome is hydrophilic interaction liquid chromatography (HILIC). Many polar and charged metabolite classes such as Ns, NTs, and amino acids can be analyzed with this stationary phase. Recently, we were also able to separate Nbs from *Arabidopsis thaliana* extracts with HILIC, as well as with a modified C18 column (our data, unpublished). Different HILIC phases and their respective potential for separating plant metabolites were excellently reviewed elsewhere [30]. The advantages of HILIC are the high tolerance to salts, the excellent reproducibility of retention times, the even peak shape, and the stability over a wide pH range, allowing the development of low-pH and high-pH methods [75]. While the low-pH methods are well suited for amino acids, vitamins, and polyamines, the high-pH protocols are preferable for carbohydrates, carbohydrate phosphates, organic acids, and NTs [76,77,78]. Additionally, nucleotide sugars can be analyzed with HILIC [48,79]. Samples for HILIC must contain a relatively high proportion of organic solvent, which is usually achieved by drying the sample and redissolving the pellet. In this process, it is important to consider the limit of solubility for the polar and charged analytes in a less polar solvent. A noisy baseline and peak tailing are sometimes observed in HILIC, especially for phosphorylated and carboxylated metabolites, due to their interaction with metallic surfaces. These problems can be addressed with deactivator additives, high-pH buffers, PEEK (polyetheretherketone)-lined columns, and PEEK-lined instruments [75,80].

Another option for the MS-compatible chromatography of polar and charged metabolites is the use of a silica-hydride stationary phase in aqueous normal-phase chromatography. These provide a high degree of polar selectivity and are also able to retain non-polar metabolites. With this approach, organic acids, NTs, and amino acids were successfully analyzed [81,82,83], but applications in plants are still scarce. However, recently, Ns from *Asparagus officinalis* extracts were successfully separated from other polar metabolites using this technique [6].

An emerging technology for the separation and detection of charged metabolites is capillary electrophoresis mass spectrometry (CE–MS) which is not yet widely in use. This technology is especially advantageous for NTs when limited sample material is available [84]. A recent study reported that the reproducibility of CE–MS among several laboratories is very good, suggesting a high potential for this technique in the future [85].

Lastly, ion chromatography (IC) using an ion exchange stationary phase is highly suited to separate cations and anions. Elution of bound analytes from ion exchange matrices usually requires a rising concentration of competing ions. Phosphate is a very popular anion classically used in anion exchange chromatography; but phosphate-based buffers are not compatible with MS. By producing potassium hydroxide as an eluent in situ via electrolysis and suppressing the eluent ions after the chromatographic separation, IC has become highly reproducible and compatible with MS. However, a comprehensive analysis of the NT/N metabolome is not straightforward with IC, because for NTs different columns and running parameters are required than for Ns. Nonetheless, NTs from plants and other organisms were successfully separated and comprehensively analyzed with IC–MS [7,86,87,88]. One challenge is that the analytes leave the instrument in a pure aqueous solution, but ionization in an electrospray ionization (ESI) source benefits from the presence of a polar organic solvent because the surface tension of pure water is too high for establishing a stable ESI spray [89]. A significant volume of organic solvent [89] or other modifiers are usually added to the analyte stream prior to the MS with a T-liquid junction. This reduces the chromatographic resolution and potentially decreases the sensitivity. Instead of a T-liquid junction, a sheath liquid interface can be used to attenuate these effects. Such an interface was employed for the analysis of sugars [90]. An alternative IC works with a weak anion exchange column and an inverse acetate gradient that is fully compatible with MS [91]. However, the stationary phase required for this technique is less durable and, currently, the availability of this matrix from commercial suppliers is limited.

## 9. Mass Spectrometry

**Figure 4:**
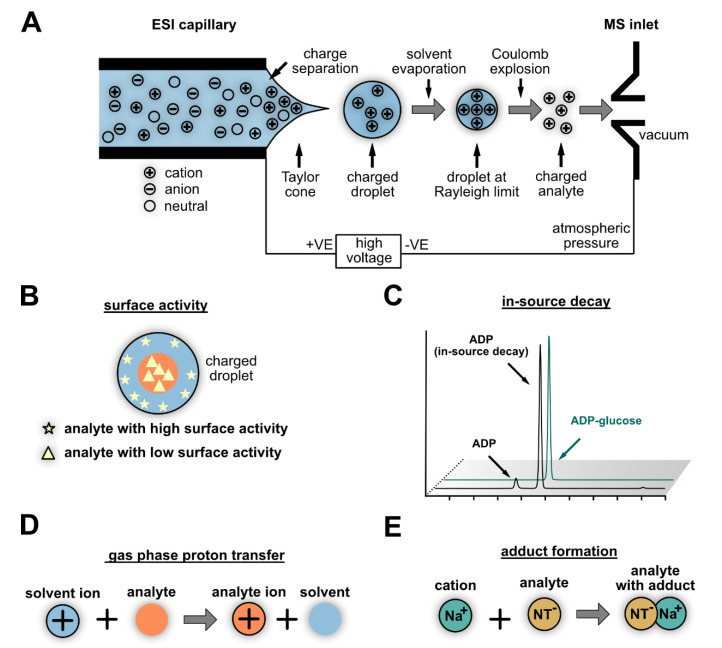
Processes in the electrospray ionization source that result in the formation of ions or influence the sensitivity of detection. (**A**) Charge separation during droplet formation and subsequent solvent evaporation results in charged analytes which can be detected by the mass spectrometer. (**B**) Analytes are not evenly distributed in the droplets. Depending on their surface activity, they locate more in the center (analytes with higher polarity; triangles) or nearer to the surface (analytes with lower polarity; stars) of the droplet. Less polar analytes with strong surface activity have a higher ionization efficiency than more polar analytes in the center of the droplet. (**C**) Decay of the analyte in the source results in signal loss. Adenosine diphosphate (ADP) is chromatographically separated from ADP-glucose in this example, but the fragmentation of ADP-glucose in the source results in the formation of ADP that appears to co-elute with ADP-glucose. If this is not noted, the amount of ADP-glucose can be underestimated (**D**). An additional process leading to ionization is the gas phase proton transfer that is determined by the proton affinities of the solvent, the modifiers, and the analytes. (**E**) The formation of adducts with, e.g., sodium or potassium, often results in the formation of two or more species from the analyte with different masses. Splitting the signal of the analyte usually leads to decreased sensitivity.

The signal strength and, thus, the sensitivity of the measurement not only depend on the abundance of the metabolite but also on (i) the ionization efficiency (IE) in the ESI source (Figure 4A), (ii) the matrix effect (ME), which is the suppression of the ionization of the target analyte by other metabolites that co-elute in the chromatography, and (iii) losses due to undesired degradation in the source (in-source decay) or inefficient fragmentation in the collision cell [89]. Other factors such as the ion transmission through the MS are also crucial for sensitivity, but they depend mostly on the technical specifications of the instrument and cannot be influenced by the operator. Polar metabolites such as NTs and Ns generally have a poor surface activity (i.e., the tendency of an analyte to be present at the surface of ESI droplets (Figure 4B)) and, therefore, have a lower ionization efficiency [89]. This results in strong ion suppression of polar metabolites by co-eluting less polar compounds [92], emphasizing the importance of selectivity in the sample preparation [93] and of resolution in the chromatography. In our analysis of NTs and Ns, we determined the ME [94] with isotope standards and concluded that the SPE significantly reduced the ME [2]. In another study, the impact of the chromatography on the ME was determined, suggesting that a separation with an HILIC stationary phase is suitable to lower the ME for polar metabolites [95].

In general, NTs can be effectively measured in the negative and positive polarity mode, but the tandem MS fragmentation spectra are more informative in the negative mode [96]. Depending on the pH of the solvent and the pKa of the metabolite, cations or anions are formed in solution. Upon charge separation (Figure 4A), these are measured in the MS in positive or negative mode, respectively [89]. Over a wide range of pH values, NTs are anions, which suggests that a detection in negative mode is the most straightforward choice. However, the negative mode requires a charge carrier in the solvent, especially when the analyte concentration is low. A mixture of hexafluoroisopropanol and methanol was used successfully as a charge carrier for the sensitive detection of oligonucleotides [97,98,99]. Additionally, 2,2,2-trifluoroethanol and formaldehyde were shown to reduce noise and increase ionization fostering a very sensitive detection of oligonucleotides [100]. One would expect that a low pH facilitates the protonation of metabolites, thus promoting the detection in the positive polarity mode, whereas a high pH results in deprotonation, boosting the charge separation of preformed anions in the negative polarity mode [89]. Interestingly, NTs are often measured at a neutral or high pH in the positive polarity mode [2,76,101,102]. This “wrong-way-round ionization” [103] also occurs for other metabolites. Ammonia, used in many mobile phases for nucleotide analysis, is hypothesized to be a gas-phase proton donor that facilitates ionization in the positive mode [89,103]. The gas-phase proton affinities, crucial for effective ionization [104], were determined for NMPs and are lower for pyrimidine NTs compared to purine NTs [105], which is consistent with our observation that, in positive polarity mode, pyrimidine NTs are detected with less sensitivity than purine NTs (our data, unpublished).

In addition to charge separation of preformed ions (Figure 4A) and gas-phase proton transfer (Figure 4D), ionization by reduction and oxidation processes, as well as adduct formation (Figure 4E), is possible. Adduct formation usually results in an altered precursor mass and a distribution of the signal between the parent ion and its adducts. This generally reduces sensitivity and complicates the interpretation of data from non-targeted approaches [106,107], although adduct formation can increase sensitivity for some carbohydrates [108]. NTs are electrostatically attracted to, e.g., sodium and potassium, and strategies to attenuate the formation of cation adducts by preconditioning the chromatographic system were shown to increase the sensitivity for the detection of oligonucleotides [106]. Moreover, the elimination of sodium originating from glassware can reduce the formation of sodium adducts [89], and adjusting the ESI source parameters (cone voltage) might disfavor adduct formation [107]. A significant amount of alkali metals can originate from the sample, and their concentration may vary depending on the environmental conditions in which the sampled plants have grown [109]. Sample preparation using an SPE approach (e.g., with a weak anion-exchange stationary phase) can potentially reduce the abundance of alkali metals in the matrix. This might be particularly important for HILIC, where NTs and Ns can co-elute with alkali metal ions. Adduct formation has been demonstrated for guanosine in HILIC [110].

Oxidation and reduction reactions in the ESI source resulting in radical formation have been described for plant metabolites [111], but it is unknown if NTs and Ns are affected by such processes. However, the oxidation of NTs can be provoked by an online electrochemical cell prior to ESI to simulate the occurrence of damaged or modified NTs [112].

In addition to adduct formation and potentially oxidation/reduction processes, the fragmentation of the NTs and Ns in the ESI source (in-source decay) can result in a loss of analyte (Figure 4C). Often, the glycosidic bond is affected [96,113], which creates the false impression that an Nb co-elutes with the corresponding N or NT (our data, unpublished, Figure 4C). A high nozzle voltage can foster in-source decay, but if this source parameter is set too low, the ionization of the analyte might be reduced [89]. In-source fragmentation can also lead to over- or underestimation of metabolite abundance, for example, when AMP, adenosine diphosphate (ADP), and ATP are not baseline-separated in the chromatography and source parameters are used that cause a decay of ATP to ADP or AMP within the source [114].

In tandem mass spectrometry, single reaction monitoring (SRM) is used, in which a precursor ion is first selected for fragmentation in the collision cell, and then the resulting product ions are quantified [115]. The value of the collision energy voltage is an important parameter for this process and needs to be optimized in the method development [116]. Interestingly, NTs exhibit characteristic fragmentation pathways that follow general rules depending on the nucleotide structure [96]. This makes the occurrence of product ions for NTs predictable, which can guide the development of an SRM assay. In addition, data on experimentally observed product ions for NTs, including many modified NT species, can be obtained from a public database [96] (www.msTide-db.com, accessed on 18 March 2021).

How do different mass spectrometers compare in performance for NT analysis? Triple-quadrupole (QqQ) instruments are traditionally considered the gold standard for the sensitive detection and quantification of metabolites. Because the mass resolution of such instruments is limited, they strongly rely on fragmentation patterns for the confident identification of Ns and NTs in a plant matrix [2,7,8,14]. Alternatively, high-resolution mass spectrometers (HRMS) provide an exact mass not only for the targeted analyte but also for all metabolites within a specified *m*/*z* (mass to charge) window, which is a tremendous advantage for non-targeted analyses and troubleshooting. In contrast to QqQ, HRMS spectra can later be reanalyzed for metabolites that were not initially in the focus of the study. With the Orbitrap and time-of-flight (TOF) mass spectrometers, two different instrument types for HRMS are available that show a similar performance for plant metabolomics [117]. Both systems can be combined with a quadrupole and a collision cell (Q-TOF and Q-Orbitrap) to select precursors and create fragment ions. For the analysis of NTs in plants, both instrument types have been used [2,12,13]. One drawback of HRMS is a reduced sensitivity compared to QqQ instruments [118]. In our recent study, we were able to detect all low-abundance dNTs in plant matrices with a QqQ instrument, but with a Q-Orbitrap some were not detectable at all or only when using the quadrupole of the Orbitrap as a narrow mass filter focusing directly on the ion mass of the dNT in question [2]. The sensitivity of an HRMS is negatively affected by the matrix. Interestingly, this not only depends mainly on ESI processes as for QqQ instruments, but also on the total ion load entering the machine at any time [118]. Reducing the ion load by selective sample preparation and good chromatographic separation is, therefore, of additional importance for sensitivity when using HR instruments. In addition to reducing the ion load, increasing the ion mass by derivatization of Ns and NTs might increase the sensitivity, because most matrix components have comparatively low *m*/*z* values, and these could be excluded by appropriate setting of the quadrupole mass filter in Q-TOF or Q-Orbitrap machines.

## 10. Outlook

Although the comprehensive quantification of Ns and NTs is now possible in samples from a variety of plants and algae, many challenges still remain. While current workflows can detect the relatively low abundant dNs and dNTs in plants [2,11], reports about the detection of damaged or modified NTs which are probably even less abundant are still scarce [119,120]. Since such rare NTs are more frequently observed in non-plant systems [61,121], it is tempting to speculate that it is the complexity of the plant matrix which hampers their detection in plants. It will be necessary to boost the sensitivity to also detect these rare NTs, which may be achieved in part by further reducing the complexity of the matrix, for example, by eliminating the more abundant rNTs [11,41]. Additionally, up-scaling of the sample amount in combination with enrichment techniques and derivatization protocols might be part of a solution.

Robust sample preparation coupled with a high sensitivity of detection will improve the analysis of NTs and Ns in cases where only little starting material is available, for example, when only a certain plant tissue is investigated. It will also be feasible to analyze plants with a particularly complex metabolome and correspondingly complex matrix (e.g., in *Viscum album* [122]).

Some rNTs and rNs but not the dNs and dNTs or the modified NTs were previously reported in studies aiming at the description of the whole (polar) metabolome [6,7]. Currently, there is a tradeoff between the depth of NT analysis on one side and the ability to comprehensively describe the entire metabolome on the other side. Coupling our sample preparation protocol for NT and N analysis [2] with protocols for the simultaneous analysis of amino acids, phytohormones, and lipids [35] may help to overcome this problem. Although the sample preparation would be more time-consuming, the preparation of a single sample may be sufficient for a more comprehensive in-depth analysis of the metabolome. Potentially, several groups of non-polar but also polar and less abundant metabolites such as phytohormones, (d)NTs, and (d)Ns could be quantified from such samples. An alternative approach uses a combination of reverse-phase and HILIC chromatography to monitor lipids and polar metabolites in one analytical run [123].

A major drawback of LC–MS techniques is that they are unable to monitor NTs in living cells and cannot assess NT concentrations in particular cells within complex tissues or in subcellular compartments. For some abundant NTs such as ATP and nicotinamide adenine dinucleotide, molecular probes were developed to monitor the in vivo concentrations within *Arabidopsis thaliana* cells and tissues [124,125]. Although such probes are currently only available for very few metabolites and are less sensitive than LC–MS (a concentration of ~160 µM can be detected with the ATP sensor, compared to a sensitivity in the picomolar range for LC–MS), these techniques can complement data obtained by LC–MS. Techniques to fractionate [126,127] or isolate subcellular compartments [128] in combination with metabolite analysis via LC–MS may prove useful for the investigation of the subcellular NT and N pools in plant cells. In the future, such fractionation and isolation techniques must gain in resolution. Especially for organelle isolation techniques, quenching strategies must be devised to effectively prevent alterations to the metabolome during organelle preparation.

## Figures and Tables

**Table 1:** Milestones in the development of a workflow for the comprehensive analysis of nucleotides, nucleosides, and nucleobases in plants.

Year	Technique	Achievement	Reference
1964	Different extraction methods compared by enzymatic assays	Identification of plant-specific problems during the extraction of phosphorylated metabolites	[5]
1972	Acid quenching combined with thin-layer chromatography	Comprehensive identification of radiolabeled nucleotide triphosphates in plants	[9]
1975	Acid quenching combined with ion-exchange LC ^a^ coupled to a UV ^b^-detector	Establishment of a workflow that allows the proper quenching of enzyme activity by acidic conditions coupled with a liquid–liquid extraction removing the acid from the extractant, allowing for chromatographic separation by ion-exchange LC	[10]
1991	Acid quenching combined with β-elimination and LC–UV	Analysis of ribo- and deoxyribonucleotides in plants by LC–UV	[11]
2010	Graphitized carbon SPE ^c^ combined with porous graphitized carbon chromatography MS ^d^	Comprehensive analysis of ribonucleotides and nucleotide sugars in plants utilizing porous graphitized carbon chromatography	[12]
2011	SPE combined with liquid chromatography and time-of-flight MS	Simultaneous analysis of 23 nucleotides and cofactors	[13]
2013	SPE combined with LC–MS	Comprehensive analysis of nucleosides and nucleobases	[14]
2020	SPE combined with LC–MS	Comprehensive analysis of nucleotides and nucleosides	[2]

^a^ liquid chromatography; ^b^ ultraviolet; ^c^ solid-phase extraction; ^d^ mass spectrometry.

## Data Availability

pKa values for many metabolites can be found in the iBond2.0 database available at http://ibond.nankai.edu.cn, 18 March 2021; fragmentation patterns for NTs can be obtained from www.msTide-db.com, 18 March 2021.

## References

[1] Witte C.-P., Herde M. (2020). Nucleotide metabolism in plants. Plant Physiol..

[2] Straube H., Niehaus M., Zwittian S., Witte C.-P., Herde M. (2020). Enhanced nucleotide analysis enables the quantification of deoxynucleotides in plants and algae revealing connections between nucleoside and deoxynucleoside metabolism. Plant Cell.

[3] Hodgson D.R.W. (2017). Physicochemical Aspects of Aqueous and Nonaqueous Approaches to the Preparation of Nucleosides, Nucleotides and Phosphate Ester Mimics. Adv. Phys. Org..

[4] Liu B., Winkler F., Herde M., Witte C.-P., Grosshans J. (2019). A Link between deoxyribonucleotide metabolites and embryonic cell-cycle control. Curr. Biol..

[5] Bielski R.L. (1964). The problem of halting enzyme action when extracting plant tissues. Anal. Biochem..

[6] Creydt M., Fischer M. (2017). Plant metabolomics: Maximizing metabolome coverage by optimizing mobile phase additives for nontargeted mass spectrometry in positive and negative electrospray ionization mode. Anal. Chem..

[7] Rolletschek H., Melkus G., Grafahrend-Belau E., Fuchs J., Heinzel N., Schreiber F., Jakob P.M., Borisjuk L. (2011). Combined noninvasive imaging and modeling approaches reveal metabolic compartmentation in the barley endosperm. Plant Cell.

[8] De Souza A.P., Cocuron J.-C., Garcia A.C., Alonso A.P., Buckeridge M.S. (2015). Changes in whole-plant metabolism during the grain-filling stage in sorghum grown under elevated CO_2_ and drought. Plant Physiol..

[9] Nygaard P. (1972). Deoxyribonucleotide pools in plant-tissue cultures. Physiol. Plant..

[10] Khym J.X. (1975). An analytical system for rapid separation of tissue nucleotides at low pressures on conventional anion exchangers. Clin. Chem..

[11] Dutta I., Dutta P.K., Smith D.W., O’Donovan G.A. (1991). High-performance liquid-chromatography of deoxynucleoside diphosphates and triphosphates in tomato roots. J. Chromatogr. A.

[12] Pabst M., Grass J., Fischl R., Léonard R., Jin C., Hinterkörner G., Borth N., Altmann F. (2010). Nucleotide and nucleotide sugar analysis by liquid chromatography-electrospray ionization-mass spectrometry on surface-conditioned porous graphitic carbon. Anal. Chem..

[13] Guerard F., Petriacq P., Gakiere B., Tcherkez G. (2011). Liquid chromatography/time-of-flight mass spectrometry for the analysis of plant samples: A method for simultaneous screening of common cofactors or nucleotides and application to an engineered plant line. Plant Physiol. Biochem..

[14] Kopecna M., Blaschke H., Kopecny D., Vigouroux A., Koncitikova R., Novak O., Kotland O., Strnad M., Morera S., von Schwartzenberg K. (2013). Structure and function of nucleoside hydrolases from physcomitrella patens and maize catalyzing the hydrolysis of purine, pyrimidine, and cytokinin ribosides. Plant Physiol..

[15] Kuskovsky R., Buj R., Xu P., Hofbauer S., Doan M.T., Jiang H., Bostwick A., Mesaros C., Aird K.M., Snyder N.W. (2019). Simultaneous isotope dilution quantification and metabolic tracing of deoxyribonucleotides by liquid chromatography high resolution mass spectrometry. Anal. Biochem..

[16] Rodríguez-González P., García Alonso J.I. (2018). Isotope Dilution Mass Spectrometry.

[17] Ingle J. (1963). The extraction and estimation of nucleotides and nucleic acids from plant material. Phytochemistry.

[18] Ullrich J., Calvin M. (1962). Alcohol-resistant phosphatase activity in chloroplasts. Biochim. Biophys. Acta.

[19] Ullrich J. (1963). Phosphatase action on phosphoglycolic, 3-phosphoglyceric, and phosphoenol pyruvic acids in spinach chloroplast fragments in the presence and absence of high concentrations of methanol. Biochim. Biophys. Acta.

[20] Ikuma H., Tetley R.M. (1976). Possible interference by an acid-stable enzyme during extraction of nucleoside diphosphates and triphosphates from higher-plant tissues. Plant Physiol..

[21] Runeckles V.C. (1958). Formation of alkyl phosphates in wheat leaves. Nature.

[22] Baccolini C., Witte C.-P. (2019). AMP and GMP catabolism in arabidopsis converge on xanthosine, which is degraded by a nucleoside hydrolase heterocomplex. Plant Cell.

[23] Dietmair S., Timmins N.E., Gray P.P., Nielsen L.K., Krömer J.O. (2010). Towards quantitative metabolomics of mammalian cells: Development of a metabolite extraction protocol. Anal. Biochem..

[24] Dahncke K., Witte C.-P. (2013). Plant purine nucleoside catabolism employs a guanosine deaminase required for the generation of xanthosine in arabidopsis. Plant Cell.

[25] Chen M., Herde M., Witte C.-P. (2016). Of the nine cytidine deaminase-like genes in arabidopsis, eight are pseudogenes and only one is required to maintain pyrimidine homeostasis in vivo. Plant Physiol..

[26] Chida J., Yamane K., Takei T., Kido H. (2012). An efficient extraction method for quantitation of adenosine triphosphate in mammalian tissues and cells. Anal. Chim. Acta.

[27] Rabinowitz J.D., Kimball E. (2007). Acidic acetonitrile for cellular metabolome extraction from Escherichia coli. Anal. Chem..

[28] Au J.L., Su M.H., Wientjes M.G. (1989). Extraction of intracellular nucleosides and nucleotides with acetonitrile. Clin. Chem..

[29] Chen H., Zhang B., Hicks L.M., Xiong L. (2011). A nucleotide metabolite controls stress-responsive gene expression and plant development. PLoS ONE.

[30] Liu Z., Rochfort S. (2014). Recent progress in polar metabolite quantification in plants using liquid chromatography-mass spectrometry. J. Integr. Plant Biol..

[31] Zbornikova E., Knejzlik Z., Hauryliuk V., Krasny L., Rejman D. (2019). Analysis of nucleotide pools in bacteria using HPLC-MS in HILIC mode. Talanta.

[32] Riondet C., Morel S., Alcaraz G. (2005). Determination of total ribonucleotide pool in plant materials by high-pH anion-exchange high-performance liquid chromatography following extraction with potassium hydroxide. J. Chromatogr. A.

[33] Brown P.R. (1971). Stability of nucleotide solutions on storage as determined by high-pressure liquid chromatography. Anal. Biochem..

[34] Barnes J., Tian L., Loftis J., Hiznay J., Comhair S., Lauer M., Dweik R. (2016). Isolation and analysis of sugar nucleotides using solid phase extraction and fluorophore assisted carbohydrate electrophoresis. MethodsX.

[35] Salem M.A., Yoshida T., de Souza L.P., Alseekh S., Bajdzienko K., Fernie A.R., Giavalisco P. (2020). An improved extraction method enables the comprehensive analysis of lipids, proteins, metabolites and phytohormones from a single sample of leaf tissue under water-deficit stress. Plant J..

[36] Soga T., Ishikawa T., Igarashi S., Sugawara K., Kakazu Y., Tomita M. (2007). Analysis of nucleotides by pressure-assisted capillary electrophoresis-mass spectrometry using silanol mask technique. J. Chromatogr. A.

[37] Cordell R.L., Hill S.J., Ortori C.A., Barrett D.A. (2008). Quantitative profiling of nucleotides and related phosphate-containing metabolites in cultured mammalian cells by liquid chromatography tandem electrospray mass spectrometry. J. Chromatogr. B.

[38] Nieman R.H., Pap D.L., Clark R.A. (1978). Rapid purification of plant nucleotide extracts with xad-2,polyvinyl-polypyrrolidone and charcoal. J. Chromatogr. A.

[39] Tanaka K., Yoshioka A., Tanaka S., Wataya Y. (1984). An improved method for the quantitative determination deoxyribonucleoside triphosphates in cell-extracts. Anal. Biochem..

[40] Uziel M. (1973). Periodate oxidation and amine-catalyzed elimination of terminal nucleoside from adenylate or ribonucleic-acid products of overoxidation. Biochemistry.

[41] Odmark G., Kihlman B.A. (1965). Effects of chromosome-breaking purine derivates on nucleic acid synthesis and on levels of adenosine 5′-triphosphate and deoxyadenosine 5′-triphosphate in bean root tips. Mutat. Res. Fundam. Mol. Mech. Mutagen..

[42] Hennere G., Becher F., Pruvost A., Goujard C., Grassi J., Benech H. (2003). Liquid chromatography-tandem mass spectrometry assays for intracellular deoxyribonucleotide triphosphate competitors of nucleoside antiretrovirals. J. Chromatogr. B.

[43] Ji P. (2016). iBonD 2.0-the Most Comprehensive pKa and BDE Database so Far.

[44] Chen X., Wu Y., Huang L., Yang L., Hong R., Yao H., Li S. (2019). Magnetic dispersive solid-phase micro-extraction combined with high-performance liquid chromatography for determining nucleotides in anoectochilus roxburghii (Wall.) Lindl. J. Pharm. Biomed. Anal..

[45] Hennion M.C. (2000). Graphitized carbons for solid-phase extraction. J. Chromatogr. A.

[46] Behmüller R., Forstenlehner I.C., Tenhaken R., Huber C.G. (2014). Quantitative HPLC-MS analysis of nucleotide sugars in Plant Cells following off-line SPE sample preparation. Anal. Bioanal. Chem..

[47] Rautengarten C., Heazlewood J.L., Ebert B. (2019). Profiling Cell Wall Monosaccharides and Nucleotide-Sugars from Plants. Curr. Protoc. Plant Biol..

[48] Ito J., Herter T., Baidoo E.E.K., Lao J., Vega-Sanchez M.E., Smith-Moritz A.M., Adams P.D., Keasling J.D., Usadel B., Petzold C.J. (2014). Analysis of plant nucleotide sugars by hydrophilic interaction liquid chromatography and tandem mass spectrometry. Anal. Biochem..

[49] Guo M., Yin D., Han J., Zhang L., Li X., He D., Du Y., Tang D. (2016). Phenylboronic acid modified solid-phase extraction column: Preparation, characterization, and application to the analysis of amino acids in sepia capsule by removing the maltose. J. Sep. Sci..

[50] Xin P., Li B., Yan J., Chu J. (2018). Pursuing extreme sensitivity for determination of endogenous brassinosteroids through direct fishing from plant matrices and eliminating most interferences with boronate affinity magnetic nanoparticles. Anal. Bioanal. Chem..

[51] Chen L., Wang X., Lu W., Wu X., Li J. (2016). Molecular imprinting: Perspectives and applications. Chem. Soc. Rev..

[52] Jadda R., Madhumanchi S., Suedee R. (2019). Novel adsorptive materials by adenosine 5ʹ-triphosphate imprinted-polymer over the surface of polystyrene nanospheres for selective separation of adenosine 5ʹ-triphosphate biomarker from urine. J. Sep. Sci..

[53] Jegourel D., Delepee R., Breton F., Rolland A., Vidal R., Agrofoglio L.A. (2008). Molecularly imprinted polymer of 5-methyluridine for solid-phase extraction of pyrimidine nucleoside cancer markers in urine. Bioorg. Med. Chem..

[54] Choi J.-H., Abe N., Tanaka H., Fushimi K., Nishina Y., Morita A., Kiriiwa Y., Motohashi R., Hashizume D., Koshino H. (2010). Plant-growth regulator, imidazole-4-carboxamide, produced by the fairy ring forming fungus Lepista sordida. J. Agric. Food Chem..

[55] Choi J.-H., Ohnishi T., Yamakawa Y., Takeda S., Sekiguchi S., Maruyama W., Yamashita K., Suzuki T., Morita A., Ikka T. (2014). The Source of “Fairy Rings”: 2-Azahypoxanthine and its Metabolite Found in a Novel Purine Metabolic Pathway in Plants. Angew. Chem..

[56] Choi J.-H., Wu J., Sawada A., Takeda S., Takemura H., Yogosawa K., Hirai H., Kondo M., Sugimoto K., Asakawa T. (2018). N-Glucosides of Fairy Chemicals, 2-Azahypoxanthine and 2-Aza-8-oxohypoxanthine, in Rice. Org. Lett..

[57] Stevens W.C., Hill D.C. (2009). General methods for flash chromatography using disposable columns. Mol. Divers..

[58] Still W.C., Kahn M., Mitra A. (1978). Rapid chromatographic technique for preparative separations with modern resolution. J. Org. Chem..

[59] Takemura H., Choi J.-H., Matsuzaki N., Taniguchi Y., Wu J., Hirai H., Motohashi R., Asakawa T., Ikeuchi K., Inai M. (2019). A Fairy Chemical, Imidazole-4-carboxamide, is Produced on a Novel Purine Metabolic Pathway in Rice. Sci. Rep..

[60] Nordstrom A., Tarkowski P., Tarkowska D., Dolezal K., Astot C., Sandberg G., Moritz T. (2004). Derivatization for LC electrospray ionization-MS: A tool for improving reversed-phase separation and ESI responses of bases, ribosides, and intact nucleotides. Anal. Chem..

[61] Jiang H.-P., Xiong J., Liu F.-L., Ma C.-J., Tang X.-L., Yuan B.-F., Feng Y.-Q. (2018). Modified nucleoside triphosphates exist in mammals. Chem. Sci..

[62] Luo X.-T., Cai B.-D., Jiang H.-P., Xiao H.-M., Yuan B.-F., Feng Y.-Q. (2019). Sensitive analysis of trehalose-6-phosphate and related sugar phosphates in plant tissues by chemical derivatization combined with hydrophilic interaction liquid chromatography-tandem mass spectrometry. J. Chromatogr. A.

[63] Bjorkman P.O., Tillberg E. (1996). Acetylation of cytokinins and modified adenine compounds: A simple and non-destructive derivatization method for gas chromatography—Mass spectrometric analysis. Phytochem. Anal..

[64] Zhang H., Li Y., Li Z., Lam C.W.K., Zhu P., Wang C., Zhou H., Zhang W. (2021). MTBSTFA derivatization-LC-MS/MS approach for the quantitative analysis of endogenous nucleotides in human colorectal carcinoma cells. J. Pharm. Anal..

[65] Dudley E., Bond L. (2014). Mass spectrometry analysis of nucleosides and nucleotides. Mass Spectrom. Rev..

[66] Salvatore F., Paul R.H., Colin F.P., Marja-Liisa R. (2017). Liquid Chromatography.

[67] Sykora D., Rezanka P., Zaruba K., Kral V. (2019). Recent advances in mixed-mode chromatographic stationary phases. J. Sep. Sci..

[68] Wilson N.S., Gilroy J., Dolan J.W., Snyder L.R. (2004). Column selectivity in reversed-phase liquid chromatography—VI. Columns with embedded or end-capping polar groups. J. Chromatogr. A.

[69] Zuvela P., Skoczylas M., Liu J.J., Baczek T., Kaliszan R., Wong M.W., Buszewski B. (2019). Column Characterization and Selection Systems in Reversed-Phase High-Performance Liquid Chromatography. Chem. Rev..

[70] Traube F.R., Schiffers S., Iwan K., Kellner S., Spada F., Mueller M., Carell T. (2019). Isotope-dilution mass spectrometry for exact quantification of noncanonical DNA nucleosides. Nat. Protoc..

[71] Gustavsson S.A., Samskog J., Markides K.E., Langstrom B. (2001). Studies of signal suppression in liquid chromatography-electrospray ionization mass spectrometry using volatile ion-pairing reagents. J. Chromatogr. A.

[72] Annesley T.M. (2003). Ion suppression in mass spectrometry. Clin. Chem..

[73] Lajin B., Goessler W. (2020). Fluoroalkylamines: Novel, highly volatile, fast-equilibrating, and electrospray ionization-mass spectrometry signal-enhancing cationic ion-interaction reagents. Anal. Chem..

[74] Dodbiba E., Breitbach Z.S., Wanigasekara E., Payagala T., Zhang X., Armstrong D.W. (2010). Detection of nucleotides in positive-mode electrospray ionization mass spectrometry using multiply-charged cationic ion-pairing reagents. Anal. Bioanal. Chem..

[75] Hsiao J.J., van de Bittner G.C., Kennedy A.P., Wei T.-C. (2018). The use of HILIC zwitterionic phase superficially porous particles for metabolomics analysis. LC GC N. Am..

[76] Kong Z., Jia S., Chabes A.L., Appelblad P., Lundmark R., Moritz T., Chabes A. (2018). Simultaneous determination of ribonucleoside and deoxyribonucleoside triphosphates in biological samples by hydrophilic interaction liquid chromatography coupled with tandem mass spectrometry. Nucleic Acids Res..

[77] Huang Y., Li W., Minakova A., Anumol T., Keller A. (2018). Quantitative analysis of changes in amino acids levels for cucumber (*Cucumis sativus*) exposed to nano copper. Nanoimpact.

[78] Kate P., John K.L. (2021). Determination of selected water-soluble vitamins (thiamine, riboflavin, nicotinamide and pyridoxine) from a food matrix using hydrophilic interaction liquid chromatography coupled with mass spectroscopy. J. Chromatogr. B.

[79] Warth B., Siegwart G., Lemmens M., Krska R., Adam G., Schuhmacher R. (2015). Hydrophilic interaction liquid chromatography coupled with tandem mass spectrometry for the quantification of uridine diphosphate-glucose, uridine diphosphate-glucuronic acid, deoxynivalenol and its glucoside: In-house validation and application to wheat. J. Chromatogr. A.

[80] Hsiao J.J., Potter O.G., Chu T.-W., Yin H. (2018). Improved LC/MS Methods for the analysis of metal-sensitive analytes using medronic acid as a mobile phase additive. Anal. Chem..

[81] Matyska M.T., Pesek J.J., Duley J., Zamzami M., Fischer S.M. (2010). Aqueous normal phase retention of nucleotides on silica hydride-based columns: Method development strategies for analytes revelant in clinical analysis. J. Sep. Sci..

[82] Pesek J.J., Matyska M.T., Fischer S.M., Sana T.R. (2008). Analysis of hydrophilic metabolites by high-performance liquid chromatography-mass spectrometry using a silica hydride-based stationary phase. J. Chromatogr. A.

[83] Pesek J.J., Matyska M.T., Loo J.A., Fischer S.M., Sana T.R. (2009). Analysis of hydrophilic metabolites in physiological fluids by HPLC-MS using a silica hydride-based stationary phase. J. Sep. Sci..

[84] Zhang W., Guled F., Hankemeier T., Ramautar R. (2020). Profiling nucleotides in low numbers of mammalian cells by sheathless CE-MS in positive ion mode: Circumventing corona discharge. Electrophoresis.

[85] Drouin N., van Mever M., Zhang W., Tobolkina E., Ferre S., Servais A.-C., Gou M.-J., Nyssen L., Fillet M., Lageveen-Kammeijer G.S.M. (2020). Capillary electrophoresis-mass spectrometry at trial by metabo-ring: Effective electrophoretic mobility for reproducible and robust compound annotation. Anal. Chem..

[86] Stafsnes M.H., Rost L.M., Bruheim P. (2018). Improved phosphometabolome profiling applying isotope dilution strategy and capillary ion chromatography-tandem mass spectrometry. J. Chromatogr. B.

[87] Schwaiger M., Rampler E., Hermann G., Miklos W., Berger W., Koellensperger G. (2017). Anion-exchange chromatography coupled to high-resolution mass spectrometry: A powerful tool for merging targeted and non-targeted metabolomics. Anal. Chem..

[88] Walsby-Tickle J., Gannon J., Hvinden I., Bardella C., Abboud M.I., Nazeer A., Hauton D., Pires E., Cadoux-Hudson T., Schofield C.J. (2020). Anion-exchange chromatography mass spectrometry provides extensive coverage of primary metabolic pathways revealing altered metabolism in IDH1 mutant cells. Commun. Biol..

[89] Cech N.B., Enke C.G. (2001). Practical implications of some recent studies in electrospray ionization fundamentals. Mass Spectrom. Rev..

[90] Xu X.-B., Liu D.-B., Guo X.M., Yu S.-J., Yu P. (2014). Improvement of sugar analysis sensitivity using anion-exchange chromatography-electrospray ionization mass spectrometry with sheath liquid interface. J. Chromatogr. A.

[91] Shi G., Wu J.T., Li Y., Geleziunas R., Gallagher K., Emm T., Olah T., Unger S. (2002). Novel direct detection method for quantitative determination of intracellular nucleoside triphosphates using weak anion exchange liquid chromatography/tandem mass spectrometry. Rapid Commun. Mass Spectrom..

[92] Cech N.B., Enke C.G. (2000). Relating electrospray ionization response to nonpolar character of small peptides. Anal. Chem..

[93] Bonfiglio R., King R.C., Olah T.V., Merkle K. (1999). The effects of sample preparation methods on the variability of the electrospray ionization response for model drug compounds. Rapid Commun. Mass Spectrom..

[94] Matuszewski B.K., Constanzer M.L., Chavez-Eng C.M. (2003). Strategies for the assessment of matrix effect in quantitative bioanalytical methods based on HPLC-MS/MS. Anal. Chem..

[95] Havlikova L., Vlckova H., Solich P., Novakova L. (2013). HILIC UHPLC-MS/MS for fast and sensitive bioanalysis: Accounting for matrix effects in method development. Bioanalysis.

[96] Strzelecka D., Chmielinski S., Bednarek S., Jemielity J., Kowalska J. (2017). Analysis of mononucleotides by tandem mass spectrometry: Investigation of fragmentation pathways for phosphate- and ribose-modified nucleotide analogues. Sci. Rep..

[97] Huber C.G., Krajete A. (2000). Sheath liquid effects in capillary high-performance liquid chromatography-electrospray mass spectrometry of oligonucleotides. J. Chromatogr. A.

[98] Apffel A., Chakel J.A., Fischer S., Lichtenwalter K., Hancock W.S. (1997). Analysis of oligonucleotides by HPLC-electrospray ionization mass spectrometry. Anal. Chem..

[99] Griffey R.H., Greig M.J., Gaus H.J., Liu K., Monteith D., Winniman M., Cummins L.L. (1997). Characterization of oligonucleotide metabolism in vivo via liquid chromatography electrospray tandem mass spectrometry with a quadrupole ion trap mass spectrometer. J. Mass Spectrom..

[100] Wu Z., Gao W., Phelps M.A., Wu D., Miller D.D., Dalton J.T. (2004). Favorable effects of weak acids on negative-ion electrospray ionization mass spectrometry. Anal. Chem..

[101] Cohen S., Megherbi M., Jordheim L.P., Lefebvre I., Perigaud C., Dumontet C., Guitton J. (2009). Simultaneous analysis of eight nucleoside triphosphates in cell lines by liquid chromatography coupled with tandem mass spectrometry. J. Chromatogr. B.

[102] Guo S., Duan J., Qian D., Wang H., Tang Y., Qian Y., Wu D., Su S., Shang E. (2013). Hydrophilic interaction ultra-high performance liquid chromatography coupled with triple quadrupole mass spectrometry for determination of nucleotides, nucleosides and nucleobases in Ziziphus plants. J. Chromatogr. A.

[103] Zhou S.L., Cook K.D. (2000). Protonation in electrospray mass spectrometry: Wrong-way-round or right-way-round?. J. Am. Soc. Mass Spectrom..

[104] Amad M.H., Cech N.B., Jackson G.S., Enke C.G. (2000). Importance of gas-phase proton affinities in determining the electrospray ionization response for analytes and solvents. J. Mass Spectrom..

[105] Green-Church K.B., Limbach P.A. (2000). Mononucleotide gas-phase proton affinities as determined by the kinetic method. J. Am. Soc. Mass Spectrom..

[106] Birdsall R.E., Gilar M., Shion H., Yu Y.Q., Chen W. (2016). Reduction of metal adducts in oligonucleotide mass spectra in ion-pair reversed-phase chromatography/mass spectrometry analysis. Rapid Commun. Mass Spectrom..

[107] Schug K., McNair H.M. (2003). Adduct formation in electrospray ionization mass spectrometry II. Benzoic acid derivatives. J. Chromatogr. A.

[108] Harvey D.J. (2000). Collision-induced fragmentation of underivatized N-linked carbohydrates ionized by electrospray. J. Mass Spectrom..

[109] Ernst M., Silva D.B., Silva R.R., Vencio R.Z.N., Lopes N.P. (2014). Mass spectrometry in plant metabolomics strategies: From analytical platforms to data acquisition and processing. Nat. Prod. Rep..

[110] Erngren I., Haglof J., Engskog M.K.R., Nestor M., Hedeland M., Arvidsson T., Pettersson C. (2019). Adduct formation in electrospray ionisation-mass spectrometry with hydrophilic interaction liquid chromatography is strongly affected by the inorganic ion concentration of the samples. J. Chromatogr. A.

[111] Vessecchi R., Crotti A.E.M., Guaratini T., Colepicolo P., Galembeck S.E., Lopes N.P. (2007). Radical ion generation processes of organic compounds in electrospray ionization mass spectrometry. Mini Rev. Org. Chem..

[112] Studzinska S., Siecinska L., Buszewski B. (2018). On-line electrochemistry/electrospray ionization mass spectrometry (EC-ESI-MS) system for the study of nucleosides and nucleotides oxidation products. J. Pharm. Biomed. Anal..

[113] Weissberg A., Dagan S. (2011). Interpretation of ESI(+)-MS-MS spectra—Towards the identification of “unknowns”. Int. J. Mass Spectrom..

[114] Xu Y.-F., Lu W., Rabinowitz J.D. (2015). Avoiding misannotation of in-source fragmentation products as cellular metabolites in liquid chromatography-mass spectrometry-based metabolomics. Anal. Chem..

[115] Kondrat R.W., McClusky G.A., Cooks R.G. (1978). Multiple reaction monitoring in mass spectrometry/mass spectrometry for direct analysis of complex mixtures. Anal. Chem..

[116] Moerlein S., Schuster C., Paal M., Vogeser M. (2020). Collision energy-breakdown curves—An additional tool to characterize MS/MS methods. Clin. Mass Spectrom..

[117] Glauser G., Veyrat N., Rochat B., Wolfender J.-L., Turlings T.C.J. (2013). Ultra-high pressure liquid chromatography-mass spectrometry for plant metabolomics: A systematic comparison of high-resolution quadrupole-time-of-flight and single stage Orbitrap mass spectrometers. J. Chromatogr. A.

[118] Kaufmann A. (2020). High-resolution mass spectrometry for bioanalytical applications: Is this the new gold standard?. J. Mass Spectrom..

[119] Chen M., Witte C.-P. (2020). A kinase and a glycosylase catabolize pseudouridine in the peroxisome to prevent toxic pseudouridine monophosphate accumulation. Plant Cell.

[120] Chen M., Urs M.J., Sanchez-Gonzalez I., Olayioye M.A., Herde M., Witte C.-P. (2018). m(6)A RNA degradation products are catabolized by an evolutionarily conserved N-6-Methyl-AMP deaminase in plant and mammalian cells. Plant Cell.

[121] Galperin M.Y., Moroz O.V., Wilson K.S., Murzin A.G. (2006). House cleaning, a part of good housekeeping. Mol. Microbiol..

[122] Senkler J., Rugen N., Eubel H., Hegermann J., Braun H.-P. (2018). Absence of complex I implicates rearrangement of the respiratory chain in european mistletoe. Curr. Biol..

[123] Schwaiger M., Schoeny H., El Abiead Y., Hermann G., Rampler E., Koellensperger G. (2019). Merging metabolomics and lipidomics into one analytical run. Analyst.

[124] Voon C.P., Guan X., Sun Y., Sahu A., Chan M.N., Gardestrom P., Wagner S., Fuchs P., Nietzel T., Versaw W.K. (2018). ATP compartmentation in plastids and cytosol of Arabidopsis thaliana revealed by fluorescent protein sensing. Proc. Natl. Acad. Sci. USA.

[125] Steinbeck J., Fuchs P., Negroni Y.L., Elsaesser M., Lichtenauer S., Stockdreher Y., Feitosa-Araujo E., Kroll J.B., Niemeier J.-O., Humberg C. (2020). In Vivo NADH/NAD^+^ Biosensing Reveals the Dynamics of Cytosolic Redox Metabolism in Plants. Plant Cell.

[126] Arrivault S., Guenther M., Florian A., Encke B., Feil R., Vosloh D., Lunn J.E., Sulpice R., Fernie A.R., Stitt M. (2014). Dissecting the subcellular compartmentation of proteins and metabolites in arabidopsis leaves using non-aqueous fractionation. Mol. Cell. Proteom..

[127] Fuertauer L., Kuestner L., Weckwerth W., Heyer A.G., Naegele T. (2019). Resolving subcellular plant metabolism. Plant J..

[128] Niehaus M., Straube H., Kuenzler P., Rugen N., Hegermann J., Giavalisco P., Eubel H., Witte C.-P., Herde M. (2020). Rapid affinity purification of tagged plant mitochondria (Mito-AP) for metabolome and proteome analyses. Plant Physiol..

